# Is Reconstructing the Right Ventricle-Pulmonary Artery Continuity Without a Conduit Worthwhile? Reevaluation in the Modern Era

**DOI:** 10.1016/j.atssr.2025.09.006

**Published:** 2025-10-06

**Authors:** Shintaro Nemoto, Hayato Konishi, Akiyo Suzuki, Takahiro Katsumata, Kanta Kishi, Noriyasu Ozaki, Yutaka Odanaka, Atsuko Ashida

**Affiliations:** 1 Department of Thoracic and Cardiovascular Surgery, Osaka Medical and Pharmaceutical University, Takatsuki, Osaka, Japan; 2 Department of Pediatrics, Osaka Medical and Pharmaceutical University, Takatsuki, Osaka, Japan

## Abstract

**Background:**

This retrospective study assessed the feasibility of our right ventricle-pulmonary artery reconstruction technique without a conduit.

**Methods:**

This technique consists of a connection of the pulmonary artery to the right ventricular incision for the posterior wall of the new route established exclusively using autologous tissue, and a transannular patch using a glutaraldehyde-treated autologous pericardium as the anterior wall incorporating a monocusp as pulmonary valve substitute using an extended polytetrafluoroethylene membrane.

**Results:**

From November 2007 to June 2024, 21 consecutive patients (median age, 15.2 months; median body weight, 8.7 kg) underwent this technique after 32 preparatory palliations in 20 patients. The diameter of the new route was less than 12 mm in 76% of patients. The left atrial appendage was interposed in the posterior wall in 15 patients (71.4%). There was 1 death due to fulminant pneumonia during the follow-up period (median, 5 years; maximum, 16.5 years). Eight patients required catheter balloon dilation after a median of 1.4 years postoperatively, with estimated free rates of initial catheter intervention of 57.4% and 38.2% after 5 and 10 years, respectively. Catheter intervention failed in 2 patients with immobile restrictive monocusps that were replaced with a new cusp after 2.5 years or a bioprosthetic valve after 15.6 years postoperatively. The estimated 5-year free reoperation rate was 92.9%.

**Conclusions:**

This technique could provide a reasonable alternative to the use of a small-diameter conduit, even in the modern era.

## Visual Abstract

**Figure:**
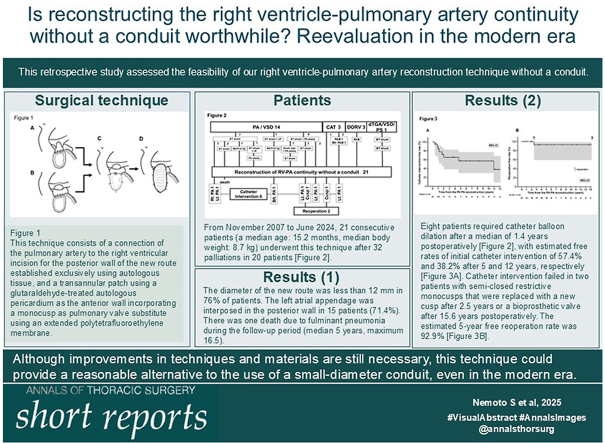

**In Short**

▪ The feasibility of our right ventricle-pulmonary artery reconstruction without a conduit was retrospectively assessed.
▪ At the expense of frequent preparatory surgery and postoperative catheter-intervention, the estimated reoperation-free rate after 5 years was 92.9%.
▪ This procedure could be a reasonable alternative to the use of a small-diameter conduit.

In order to avoid reoperation due to outgrowth or material deterioration of the pulmonary conduit (PC), various procedures using autologous tissue without a PC for reconstruction of right ventricle to pulmonary artery (RV-PA) continuity were actively practiced a few decades ago.^1^, ^2^, ^3^, ^4^ However, because various new PCs are emerging in the current era, RV-PA reconstruction without a PC has been abandoned in many centers. On the other hand, since durability of small-diameter PCs are still not satisfied,^5^, ^6^, ^7^ reconstruction using autologous tissue without a PC may still be a useful option. After confirming the effectiveness of this surgical alternative without the use of a PC for common arterial trunk,^8^ we expanded its application with technical modifications.

The subsequent retrospective study assessed the feasibility of this procedure without the need for PC by describing the surgery-related details and the long-term results.

## Patients and Methods

### Ethics Statement

The institutional review board of Osaka Medical and Pharmaceutical University approved this retrospective observational study on March 29, 2022 (#2021-183) and extended to March 31, 2025. The board waived informed consent from the patient or guardian.

### Reconstruction of the RV-PA Continuity Without a Conduit

**Figure 1:**
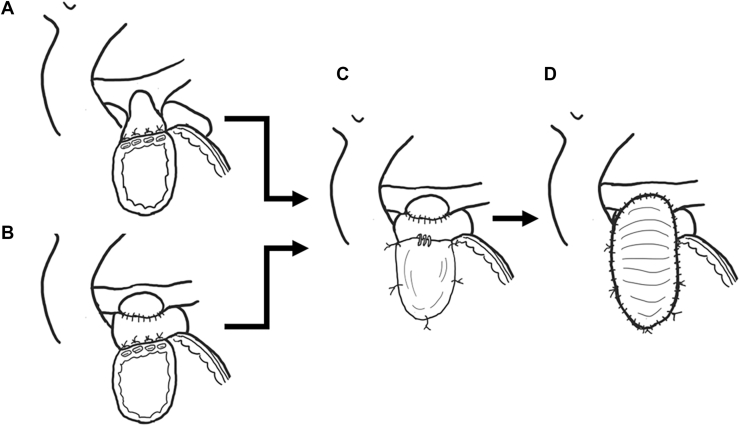
Surgical procedure to reconstruct right ventricle to pulmonary artery continuity without a conduit. (A) Direct connection between the proximal stump of the central pulmonary artery and the upper border of the right ventriculotomy. (B) Interposition of the left atrial appendage between the pulmonary artery and ventriculotomy when necessary. (C) A valve substitute using an expanded polytetrafluoroethylene membrane attached to the ventriculotomy. (D) An anterior wall of the new right ventricle to the pulmonary artery connection using glutaraldehyde-treated autologous pericardium as a transannular patch. The arrows indicate the steps of this procedure.

**Supplementary Figure 1:**
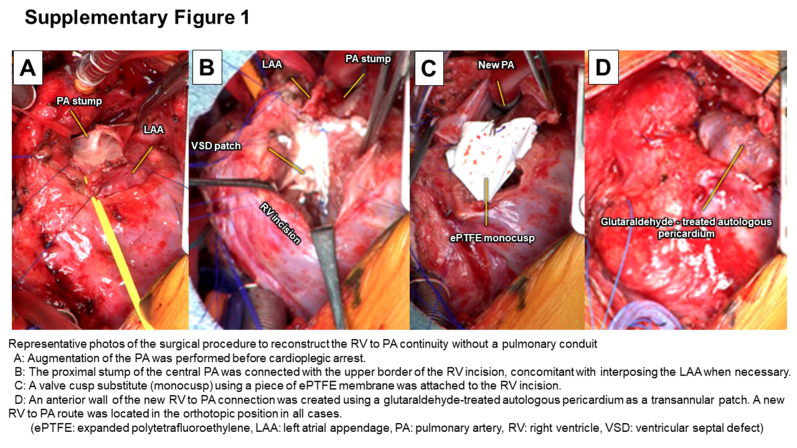


**Supplementary Figure 2:**
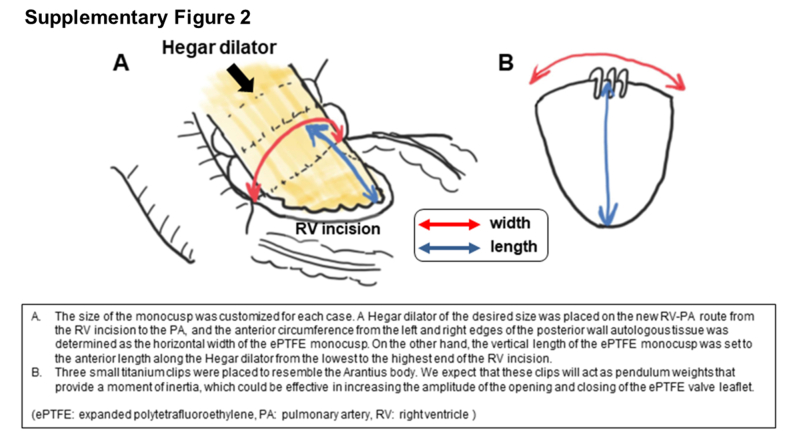


With necessary augmentation of the pulmonary artery (PA) branches, the proximal stump of the central PA was connected with the upper border of the right ventricle (RV) incision in the orthotopic position, concomitant with interposing the left atrial (LA) appendage when necessary (Figures 1A, 1B; Supplemental Figure 1). An anterior wall of the new RV-PA route was created using a glutaraldehyde-treated autologous pericardium with a monocusp using an expanded polytetrafluoroethylene (ePTFE) membrane (WL Gore & Associates, Inc), in all cases as previously described (Figures 1C, 1D; Supplemental Figures 1, 2).^8^ The diameter of the new RV-PA route was initially set to Z-score = 0, and +1-2 mm if possible. All patients received aspirin at a dose of 3-5 mg/kg daily for 3 months after surgery.

### Patient Inclusion

Consecutive patients who underwent this technique between November 1, 2007, and June 30, 2023, were included.

### Data Collection and Outcome

The series of clinical course, preoperative and perioperative categorical variables and covariates, details of surgical procedures, postoperative death and invasive reintervention, and cardiac ultrasound measurements were collected from the medical records of our institution and by contacting the local pediatricians. Outcomes were defined as death from any cause and reintervention.

### Statistics

Descriptive statistics were used to determine the overall clinical characteristics. Kaplan-Meier analysis was used to evaluate long-term outcomes after discharge using JMP Pro 16 statistical software (SAS Institute Inc).

## Results

### Patient Background

**Figure 2:**
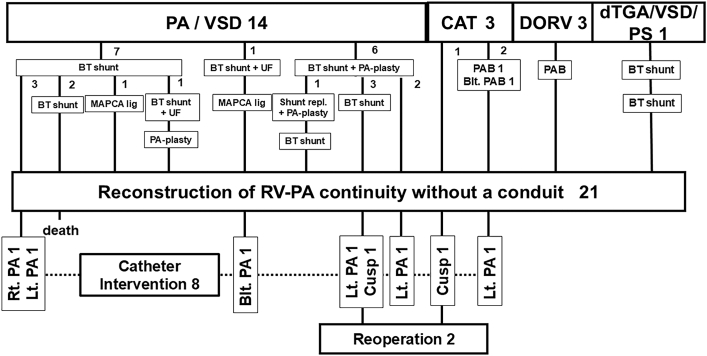
Preparatory palliative surgery performed before right ventricle to pulmonary artery reconstruction without a conduit in each main cardiac diagnosis (upper half) and postoperative catheter intervention (lower half). The numbers indicate the number of cases. (Blt.: bilateral; BT, Blalock-Taussig; CAT, common arterial trunk; DORV, double-outlet right ventricle; dTGA/VSD/PS, d-transposition of the great arteries with ventricular septal defect and pulmonary stenosis; Lt., left; MAPCA, major aortopulmonary collateral artery; PAB, pulmonary artery banding; PA-plasty, angioplasty of the pulmonary artery; PA/VSD, pulmonary atresia with ventricular septal defect; PTA, persistent truncus arteriosus; Rt., right; RV-PA, right ventricle to pulmonary artery; UF, unifocalization.)

Twenty-one consecutive patients (13 female) underwent this modification at a median age of 15.2 (interquartile range [IQR], 10-24.8) months with a median body weight of 8.7 kg (IQR 7.3 – 10.3). The main cardiac diagnosis is shown in Figure 2.

### Palliative Surgery Before RV-PA Reconstruction

In total, 32 preparatory surgeries in 20 patients were performed in advance (Figure 2). There were 10 cases that required multiple preparatory surgeries (twice: 8 cases, 3 times: 2). The median interval from last preparatory surgery to RV-PA reconstruction was 9.5 (IQR, 7.6-14.5) months.

### Operative Description

The interposition of the LA appendage was performed in 15 patients (71.4%) (a representative postoperative angiogram, 2 years after this procedure performed at age 10 months can be found as the Supplemental Video). Concomitant patch augmentation of the PA branch using glutaraldehyde-treated autologous pericardium was required in 15 patients.

A median diameter of the new RV-PA route was 12 (IQR, 12-12; min-max, 10-20) mm. Although our first choice is a hand-made ePTFE conduit when necessary, there was no intraoperative conversion to conduit placement.

### Mortality

All patients were followed up for a median period of 5 (IQR, 2.1-10.3; maximum 16.5) years. Only 1 death occurred: 34 days after surgery in a 2-year-old girl with pulmonary atresia with ventricular septal defect (PA/VSD) associated with 22q11.2 deletion. Although the patient was discharged in good condition, autopsy revealed rapid fulminant bronchopneumonia against a background of pulmonary hypertension and chronic RV myocardial impairment, even without any problems at the surgical site. The Kaplan-Meier estimated survival rate at 5 years was 95.8% (95% CI, 70.%-99.3%).

### Catheter Intervention

**Figure 3:**
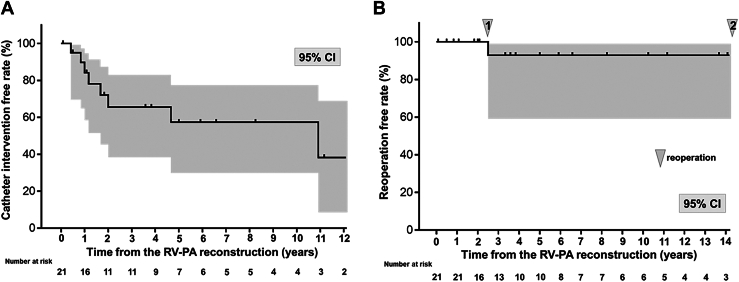
Kaplan-Meier estimates showing the free rate from reintervention after right ventricle to pulmonary artery (RV-PA) reconstruction without a conduit. (A) Free rate from catheter intervention. (B) Free rate from reoperation.

**Supplementary Figure 3:**
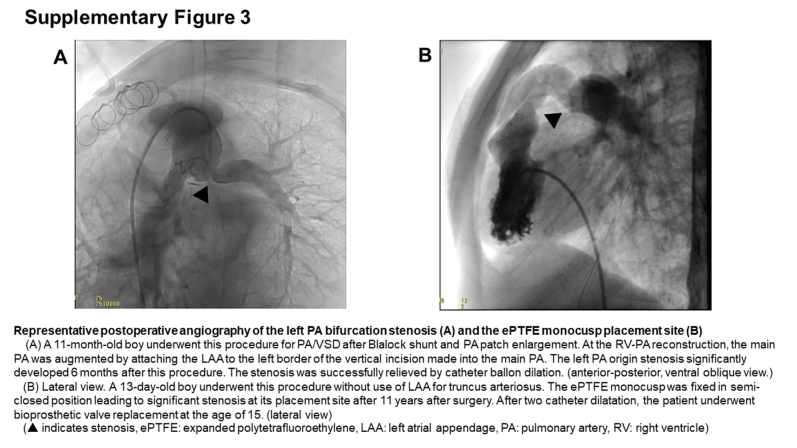


Catheter balloon dilation was required after a median of 1.4 (IQR, 1-2.7) years postoperatively (Figure 2) for 8 significant stenotic lesions in the PA branch origin at juncture to the main PA (left 4, right 1, both 1) (Supplemental Figure 3A) or at the monocusp site (2 patients) (Supplemental Figure 3B). Catheter intervention was successful except in the latter 2 patients. The Kaplan-Meier estimated free rates from the initial catheter intervention were 57.4% (95% CI, 30.0%-77.5%) and 38.2% (95% CI 8.4%-68.9%) after 5 and 10 years, respectively (Figure 3A).

### Reoperation

The monocusp embedded within the proliferated pseudointima was replaced in the above 2 cases: with a monocusp after 2.5 years for PA/VSD or a prosthetic valve after 15.6 years for common arterial trunk. Thus, the Kaplan-Meier estimated free rate from reoperation at 5 years was 92.9% (95% CI, 59.1%-99.0%) (Figure 3B).

No infective endocarditis was observed with the newly created RV-PA route throughout the observation period.

### Cardiac Ultrasound Measurement of the Monocusp Function During Postoperative Follow-up

**Figure 4:**
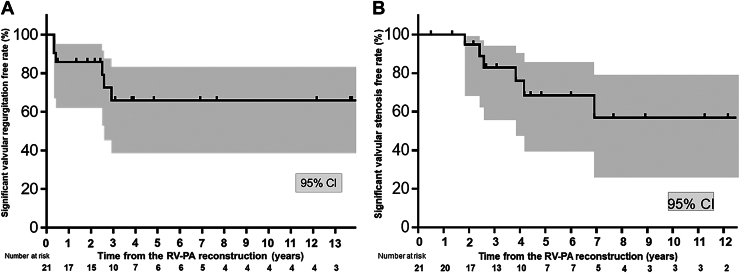
Kaplan-Meier estimates showing the free rate from significant malfunction of the expanded polytetrafluoroethylene (ePTFE) monocusp in pulmonary valve position in right ventricle to pulmonary artery (RV-PA) reconstruction without a conduit. Analysis was performed on all 21 surgeries, excluding 1 death and including 1 reoperation. (A) Free rate from moderate-severe valve regurgitation. (B) Free rate from valve stenosis of >3.0 m/s.

The Kaplan-Meier estimated free rate from moderate-severe valvular regurgitation at postoperative 2 and 5 years was 85.7% (95% CI, 60.0%-95.2%) and 65.9% (95% CI, 38.5%-83.4%), respectively (Figure 4A). The free rate from the valvular stenosis of >3.0 m/s at the same postoperative period was 94.7% (95% CI, 68.1%-99.2%) and 68.4% (95% CI, 39.2%-85.7%), respectively (Figure 4B).

## Comment

The major findings of this retrospective study are as follows. First, although 76.2% of the RV-PA reconstructions without a PC involved small diameters of 12 mm or less, the long-term reoperation-free rate was 92.9% over 5 years after this procedure. Second, preparatory surgeries, concomitant augmentation of the peripheral PA with RV-PA reconstruction, and postoperative catheter intervention were frequently required in PA/VSD. Third, although it was difficult to observe the change in the diameter of the new RV-PA route, the insufficiency and stenosis of the monocusp progressed over time, resulting in 2 reoperations.

RV-PA reconstructions without a PC have been performed for decades. Although various materials have been used in different patient populations, similar techniques have shown excellent long-term reoperation-free rates.^1^, ^2^, ^3^, ^4^ We have been using cross-linked autologous pericardium in the hope of avoiding material failure due to pseudointimal thickening and calcification when using foreign materials.^8^ The common component of these techniques, including ours, is the maximum use of PA tissue as the posterior wall of the new RV-PA route, which may be the only portion that grows and is the key to success.

From this viewpoint, it is essential to secure sufficient PA development to create an RV-PA route of sufficient diameter. Particularly in cases of PA/VSD, the main to central PA is often diminutive due to infiltration of ductal tissue or coexistence of multiple major aortopulmonary collateral arteries; preparatory palliative surgery is often required to promote PA development. Contrary to expectations, palliative surgery alone could not accomplish sufficient growth of the central part of the PA in 15 cases in which interposition of the LA appendage was required for the RV-PA posterior connection. Although the preexisting PA branch lesions remained as a risk of reintervention even after this operation, the beneficial reintervention-free rate for the central portion of RV-PA route may have been achieved by the preparatory surgeries and the LA appendage interposition. However, our policy may have delayed the timing of corrective surgery and increased the risk of development of pulmonary hypertension due to long-standing high pulmonary blood flow. In contrast to PA/VSD, in other diseases where there exists sufficient PA tissue, this procedure was performed without difficulty.

Because of the difficulty of echocardiographic observation due to the patients’ physical growth, we were unable to present the possibility of the growth of a new RV-PA route based on changes in diameter in this study. On the other hand, in the area created with autologous tissue, no stenosis occurred due to patients’ outgrowth or material degeneration. This is an advantage that cannot be expected from existing conduits. Unfortunately, 2 reoperations occurred due to failure of the ePTFE monocusp, which was embedded in the thickened pseudointima and fused with the anterior pericardium in a semiclosed position in both cases. Extensive calcification developed in one of the excised monocusp after postoperative 15.6 years.^9^ The clinical application of ideal valve substitute materials is eagerly awaited.

### Limitations

This retrospective study included a limited number of patients from a single institution. In addition, this study did not perform any comparisons, such as with vs without a conduit, or factor analysis of reoperation. To validate the results of this study, additional cases or prospective comparative studies are required.

### Conclusions

Even in the modern era, RV-PA reconstruction without a conduit is a possible alternative to small-diameter conduits to reduce the risk of reoperation.

## References

[1] Barbero Marcial M., Tamanati C. (1999). Alternative non-valved technique for repair of truncus arteriosus: long-term results. Semin Thorac Cardiovasc Surg Pediatr Card Surg Annu.

[2] Isomatsu Y., Shin’oka T., Aoki M. (2004). Establishing right ventricle-pulmonary artery continuity by autologous tissue: an alternative approach for prosthetic conduit repair. Ann Thorac Surg.

[3] Derby C.D., Kolcz J., Gidding S., Pizarro C. (2008). Outcomes following non-valved autologous reconstruction of the right ventricular outflow tract in neonates and infants. Eur J Cardiothorac Surg.

[4] Fan C., Yang Y., Xiong L. (2017). Reconstruction of the pulmonary posterior wall using in situ autologous tissue for the treatment of pulmonary atresia with ventricular septal defect. J Cardiothorac Surg.

[5] Karamlou T., Blackstone E.H., Hawkins J.A. (2006). Can pulmonary conduit dysfunction and failure be reduced in infants and children less than age 2 years at initial implantation?. J Thorac Cardiovasc Surg.

[6] Vitanova K., Cleiziou J., Hoerer J. (2014). Which type of conduit to choose for right ventricular outflow tract reconstruction in patients below 1 year of age?. Eur J Cardiothorac Surg.

[7] Yamamoto Y., Yamagishi M., Maeda Y. (2019). Histopathological analysis of expanded polytetrafluoroethylene-valved pulmonary conduit. Semin Thorac Surg.

[8] Nemoto S., Ozawa H., Sasaki T. (2011). Repair of persistent truncus arteriosus without a conduit: Sleeve resection of the pulmonary trunk from the aorta and direct right ventricle-pulmonary anastomosis. Eur J Cardiothorac Surg.

[9] Konishi H., Suzuki A., Katsumata T., Fujisawa Y., Motoyoshi T., Nemoto S. (2025). Characterization of calcium deposition on expanded polytetrafluoroethylene membrane as a valve substitute in pulmonary position. Interdiscip Cardiovasc Thorac Surg.

